# Electron-Phonon Coupling as the Source of 1/f Noise in Carbon Soot

**DOI:** 10.1038/s41598-018-36544-4

**Published:** 2019-01-30

**Authors:** M. Mihaila, D. Ursutiu, I. Sandu

**Affiliations:** 1 National Institute of Research and Development in Microtechnologies-IMT Bucharest, Erou Iancu Nicolae str. 126A, 077190 Bucharest, Romania; 2 University Transilvania - Brasov, Department of Electrical Engineering and Computer Science, Oltenia str. 2A, 500209 Brasov, Romania; 3 National Institute for Lasers, Plasma and Radiation Physics, Bucharest-Magurele 409, Atomistilor str., Magurele, 077125 Romania

## Abstract

Two 1/f noise peaks were found in a carbon soot resistor at voltages characteristic of Kohn anomalies in graphite. The ratio of the electron-phonon coupling matrix elements at the anomalies calculated from the noise peak intensities is the same as the one obtained from the Raman frequencies. This demonstrates that the electron-phonon coupling is the microscopic source of 1/f noise in carbon soot. A new, very general formula was deduced for the frequency exponent, wherein nonlinearity and dispersion are the only ingredients. The interplay between nonlinearity and dispersion in this formula describes the sublinear-supralinear transitions experimentally observed at both anomalies in the voltage dependence of the frequency exponent. A quadratic dependence of the 1/f noise parameter on the matrix element is proposed and applied to explain the M-shape of the 1/f noise in graphene. We found that the frequency exponent mimics the dependence of the noise intensity in the whole voltage range, while both are the image of the graphite phonon spectrum. This implies that the source of nonlinearity is in the electron-phonon coupling which modulates the slope of the spectrum. It requires the presence of 1/f noise in the thermal noise background of the resistor till phonon frequencies.

## Introduction

1/f noise was discovered in a vacuum tube by Johnson^1^. A decade later, it was observed in platinum films^2,3^ and granular graphite^4^. Innumerable works performed since revealed its omnipresence in solid and solid-state devices^5–19^. More recently, it was found in Van der Waals materials^17,19–21^ and nanodevices for quantum computing^18^, where it represents the main intrinsic source of decoherence. It is thus of great practical importance to find its microscopic origin, which, in spite of more than 90 years of investigations, remained unknown. The first theoretical attempts to understand where this noise comes from were made by Schottky^22^ and, latter, by Brillouin^23^, who predicted that the noise intensity should be inversely proportional to the total number of electrons (N) in the investigated resistor. In his pioneering experimental work, Bernamont stated that “this fluctuation was due to fluctuations in the number of free electrons in the conductor which would give rise to fluctuations in the resistance”^3^. The idea of number fluctuations got microscopic support when McWhorther proposed tunneling into the surface states as mechanism of carrier number fluctuations, usually known as the number fluctuation model^24^, which has been largely accepted and applied especially in the case of semiconductors and semiconductor devices^5,7,15^. In this context, Hooge reported^25^ that existing experimental 1/f noise data for both metals and semiconductors follows a 1/N rule and stated that this size effect argues against the surface origin of 1/f noise, which is a bulk phenomenon. He speculated that 1/f noise might be related to thermal noise by the influence the drift velocity could have on the carriers Brownian motion and used an empirical procedure of thermal noise renormalization to gather the 1/N factor, the noise intensity (S_V_) of the voltage (V) across the resistor terminals and the frequency (f) in the simple phenomenological formula: S_V_/V^2^ = α/Nf, where α is an empirical parameter with no physical significance. Experiments done on semiconductor samples with different doping showed that α∼(μ/μ_L_)^2^, where μ is the actual mobility dominated by impurity scattering and μ_L_ is the value of mobility when only lattice scattering would exist in the sample^26^. Since μ and, hence, α decreased with the doping, it was concluded that the microscopic source of 1/f noise is the mobility fluctuations due to phonon scattering. Consequently, α was called mobility fluctuation 1/f noise parameter and, in spite of its obscure origin, it is thereafter used as a measure of noisiness of solid state systems and devices.

The mechanism of mobility fluctuation was able to explain 1/f noise in thermo emf ^27^ and Hall voltage^28^ of intrinsic and extrinsic semiconductors. An indirect support for phonon participation in 1/f noise came from the very low noise observed in near ballistic GaAs diodes^29^ and short carbon nanotubes in the ballistic regime^30^, which was attributed to the near-absence of phonon scattering. Also, bulk and surface phonon number fluctuations have been invoked as explanation for the 1/f noise found in the light scattering by quartz crystal^31^ and current fluctuations in STM^32^, respectively. For about four decades, peaks occurring at the phonon energies were reported in the 1/f noise of different solid-state physical systems, such as GaAs-tunnel diode^33^, bipolar transistors^34^, metallic point contacts^35,36^, continuous^37^ and discontinuous platinum films^38^, quartz crystals^39^, carbonic materials^40^ and metallic carbon nanotubes^41^. Recently, silicon radiation detectors with reduced 1/f noise level were obtained by control of electron-phonon interaction at Si-SiO_2_ interface^42^. In some of these materials^38^, the noise peaks were found to be coincident with the van Hove singularities either in the phonon density of states, F(ω), or the Eliashberg function, g^2^(ω)F(ω), where g^2^(ω) is the electron-phonon matrix element and ω is the phonon frequency^38^. These observations raised the question whether the matrix element contributes or not to the noise enhancement at the phonon energies. In this respect, in metallic carbon nanotubes a single resonance-like noise peak observed by Back *et al*.^41^ close to 200 mV was attributed to the “manifestation of Kohn anomaly”^41^ at Г point of symmetry in 1/f noise. The authors found that the noise intensity factor (A = α/N) tracks the full width at the half maximum of the G-band, which indirectly points to the implication of the electron-phonon coupling in the mechanism of 1/f noise. No other experiment exists so far to definitely settle the role of electron-phonon coupling (EPC) in the 1/f noise generation.

In this work, we report 1/f noise measurements in a simple carbon soot resistor as a function of voltage. We found that 1/f noise intensity shows a fine structure, with two dominant, sharp noise peaks located at voltages corresponding to the two well-known Kohn anomalies^43^ in graphite at zone-boundary K-point and zone-center Г-point^44^, respectively. Piscanec *et al*.^44^ demonstrated that at graphite Kohn anomalies, the slope of the phonon dispersion “is proportional to the square of the electron-phonon coupling”^44^. Corroborating this property with the sharp noise increase observed at both Kohn anomalies, we realized that such a kink in the phonon dispersion can be a precious, unique test vehicle to investigate whether EPC is acting as microscopic source of 1/f noise. To this goal, we exploited the relation established by Piscanec *et al*.^44^ between the electron-phonon matrix elements at the two anomalies in graphite to calculate their ratio from two very different sets of data: Raman spectrum and noise measurements. The ratio was found the same, which imposes the electron-phonon coupling as the microscopic source of 1/f noise in carbon soot. A few ways of how to verify the validity of this result for other materials and solid-state devices are suggested. Hereafter, we associate the noise peaks with the nonlinear dissipation mechanisms and using an older theory^45^ nonlinearity and dispersion are identified as factors influencing both the 1/f noise parameter and the frequency exponent. A new, very general formula was found for the frequency exponent. Nonlinearity is identified as *sine qua non* condition for the existence of 1/f noise in both equilibrium and nonequilibrium. Analogies between the new formula and the Dutta-Dimon and Horn (DDH)^46^ equation for the spectral exponent reveal that they are closely related. The interplay nonlinearity-dispersion in this formula predicts the existence of sublinear-supralinear transitions in the exponent at Kohn anomalies, transitions which have been observed experimentally. We show that the deviations of the frequency exponent from 1 are nonlinearity signatures.

Based on these results, we propose that 1/f noise parameter is the image of the matrix element squared and use this dependence to explain the puzzling M-shape of the 1/f noise in graphene in terms of electron-phonon coupling. An unexpected, fine structure in the frequency exponent is brought to the fore. It tracks the one existing in the noise intensity in the whole voltage range, not only at anomalies. We report that some weaker peaks in both noise intensity and exponent, located aside the Kohn anomalies, strongly correlate with the specific energies of the Z phonons, the out-of-plane atomic motion in graphite (graphene). It helps unravel the same electron-phonon interaction as the unique source responsible for the structure in both noise intensity and the frequency exponent. We consider that the slope modification by electron-phonon coupling is an effect generated by the extension of the 1/f noise spectrum in thermal noise, a fundamental property of the 1/f noise which, so far, has been established only for systems in thermal equilibrium^47,48^.

## Experimental

**Figure 1:**
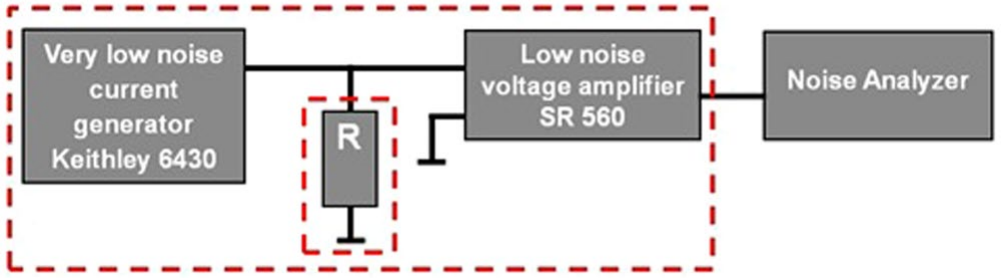
Schematic of the noise measurement system; R – carbon soot resistor; red dashed boxes – Faraday cages.

A toluene carbon soot solution was dropped between two gold contacts (100 nm thick, predeposited on a SiO_2_/Si substrate), separated by a gap of about 4.5μm. The soot, prepared by laser pyrolysis, was a mixture of nanoparticles with altered turbostratic structure and small graphitic plaques, all embedded in an amorphous carbon mass. The Raman spectrum of the soot was measured at 785 nm. The noise measurement system is presented in Fig. 1. It consists of a very low-noise current generator (Keithley 6430) which was used to inject current into the resistor. The voltage developed across the resistor terminals has been amplified (SR 560) and Fourier transformed. I − V characteristic of the film, noise spectrum and its slope (γ) have been measured from 100 mV to 300 mV, at a voltage bin of 5 mV. 15 snap-shot spectra were averaged to get the final spectrum at each voltage. All measurements were done at room temperature.

## Results and Discussion

**Figure 2:**
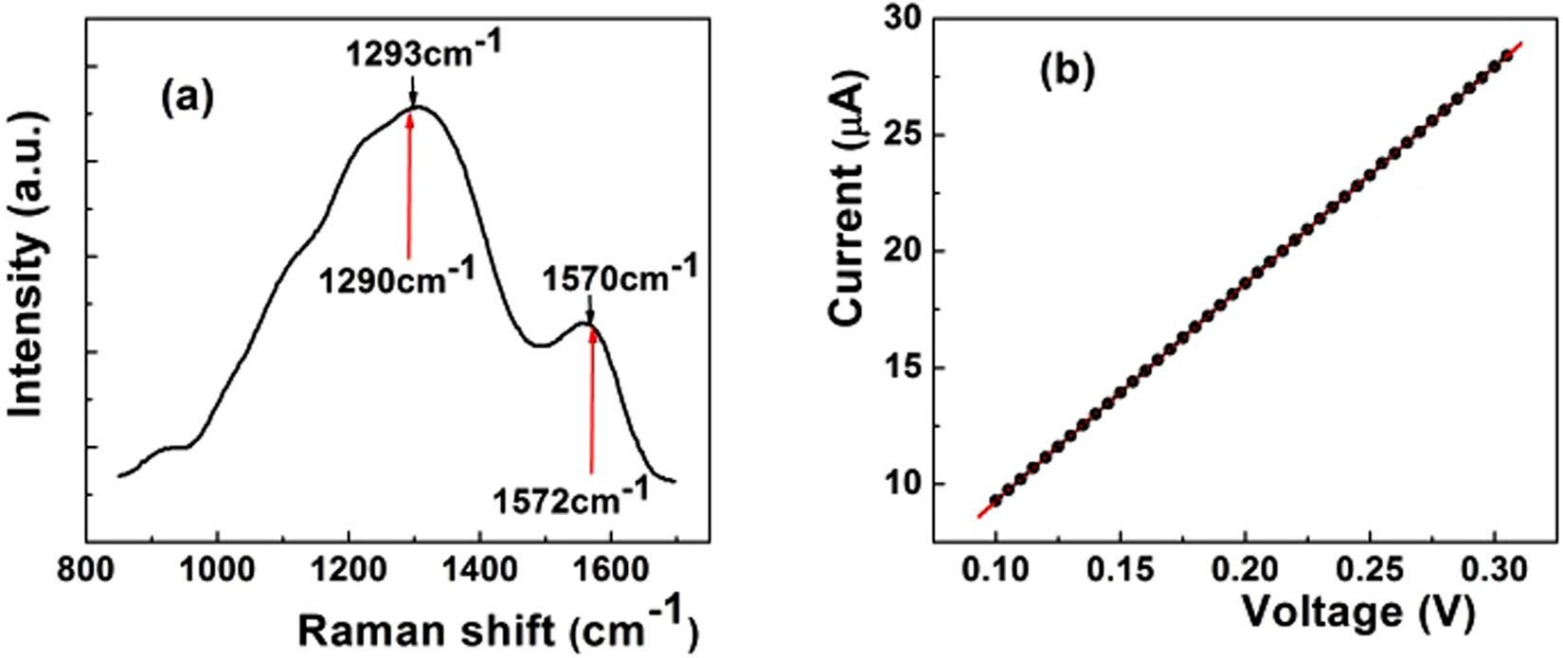
(**a**) Raman spectrum of the carbon soot. The black arrows denote the position of the D- and G Raman band, respectively, while the red ones indicate the position (in wavenumbers) of the dominant noise peaks (**b**) – I − V characteristic of the carbon soot resistor (black dots are experimental data, red line is guide to the eyes).

**Figure 3:**
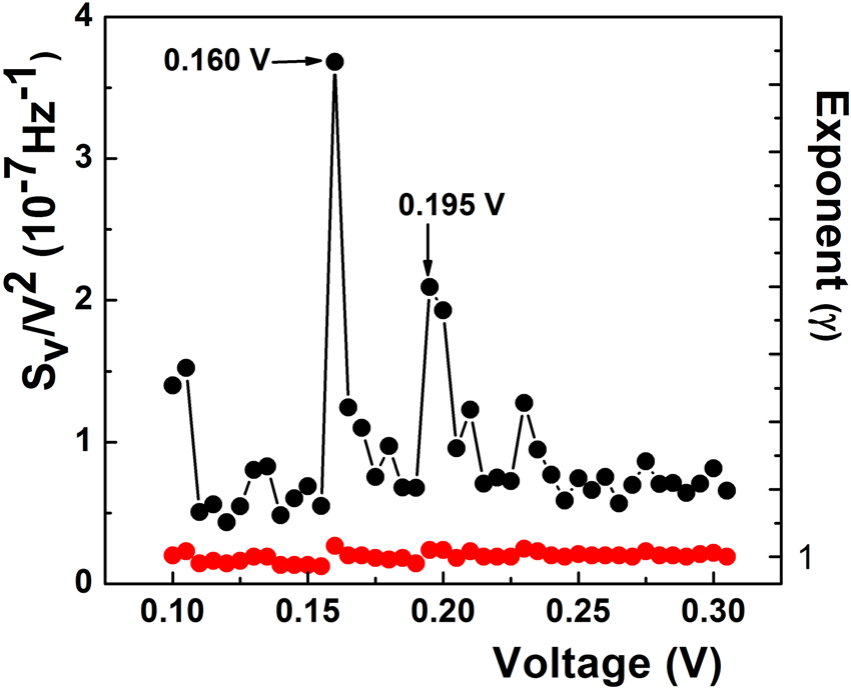
Dependences of S_V_/V^2^ (black dots) at f = 3 Hz and the exponent (γ, red dots) on voltage. The two dominant noise peaks and their voltage location are indicated by arrows.

The Raman spectrum of the soot (Fig. 2a) features two large bands at about 1293 cm^−1^ and 1570 cm^−1^, respectively. Although red-shifted in comparison with the pyrolytic graphite (1330 cm^−1^), the 1293 cm^−1^ band is associated with the D band of graphite^49^. The band at 1570 cm^−1^ is close to 1575 cm^−1^, a value attributed to the E_2g_ phonon mode^50^, the Г- point LO phonon, known as the G band of graphite^51^. Figure 2b shows that the I-V characteristic of the resistor is linear. The voltage fluctuations across the resistor terminals exhibited a 1/f^γ^ spectrum, with variations (±0.1) of γ(V) around 1, as shown in Fig. 3. Since the resistor linearity requires a quadratic dependence of S_V_ on voltage (S_V_~V^2^), the normalized noise intensity (S_V_/V^2^) should be independent on voltage. Figure 3 shows that this is not the case, for S_V_/V^2^ vs. V exhibits peaks which stand for the local violation of the Ohm law. This deviation from the linear response theory seems to be driven by microscopic nonlinearities manifesting preferentially at some voltages, as in the case of the two dominant noise peaks located at 0.160 V and 0.195 V. Both noise peaks develop at voltages corresponding to the frequencies of the optical phonons responsible for the D- and G bands of graphite at K (161 meV) and Г (196 meV) point of symmetry^49–51^, respectively. On an energy scale (1 meV = 8.06 cm^−1^), 160 mV and 195 mV correspond to 1290 cm^−1^ and 1572 cm^−1^. As shown in Fig. 2a, both values are located at the peak of the D- and G Raman band, respectively.

Since optical phonons responsible for the D- and G-band in graphite exhibit Kohn anomalies^43^ at K and Г point of symmetry^44^, respectively, we supposed that the most intense noise peaks in Fig. 3 could be Kohn anomaly-related. If one assumes that EPC would act the microscopic source of the noise peaks at Kohn anomalies in our carbonic material, the difference in the peak intensities could be tentatively assigned to the stronger coupling at the K point^44,51,52^. In search for a quantitative support of this hypothesis, we resorted to Piscanec *et al*.^44^ who demonstrated that on graphite Fermi surface, the EPC matrix elements $\langle {{\rm{g}}}_{{\rm{\Gamma }}}^{2}\rangle$ and $\langle {{\rm{g}}}_{{\rm{K}}}^{2}\rangle$ at Г and K, respectively, are connected by the relation:

1 $$(\langle {{\rm{g}}}_{{\rm{K}}}^{2}\rangle {{\rm{\omega }}}_{{\rm{K}}})/(\langle {{\rm{g}}}_{\Gamma }^{2}\rangle {{\rm{\omega }}}_{\Gamma })=2.$$

**Figure 4:**
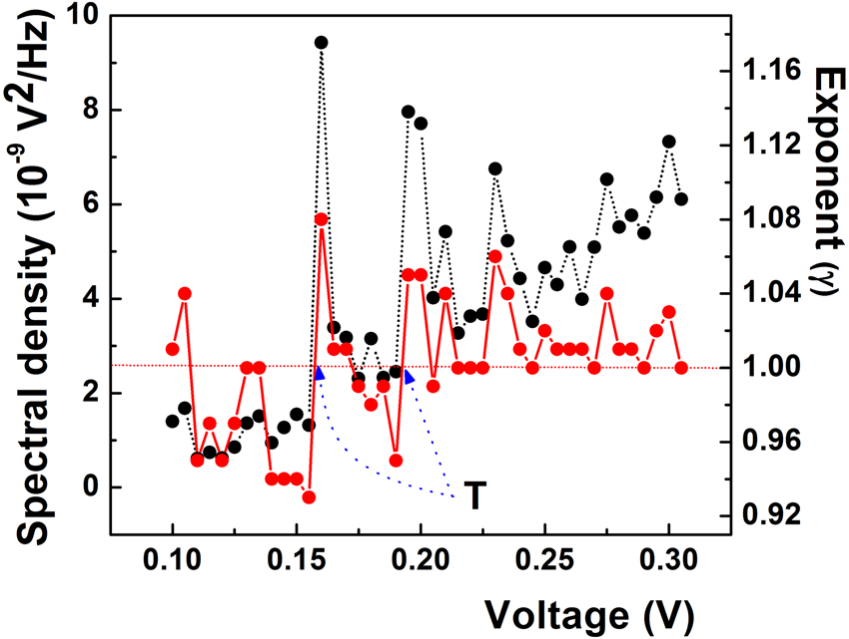
Dependences of the absolute spectral density (black dots) and the frequency exponent (γ, red dots) on voltage. Transitions (T) from γ < 1 to γ > 1 are visible at both Kohn anomalies. The two variables feature a similar structure.

With the Raman frequencies from Fig. 2a at Г (ω_Γ_ = 1570 cm^−1^) and K (ω_K_ = 1293 cm^−1^), the EPC matrix element ratio at the Kohn anomalies calculated with the relation (1) is: $\langle {{\rm{g}}}_{{\rm{K}}}^{2}\rangle /\langle {{\rm{g}}}_{{\rm{\Gamma }}}^{2}\rangle =2.428$. On the other hand, the ratio of normalized noise intensity at V_D_ = 160 mV and V_G_ = 195 mV extracted from Fig. 3 is (S_VD_/S_VG_)$({{\rm{V}}}_{{\rm{G}}}^{2}/{{\rm{V}}}_{{\rm{D}}}^{2})$ = 1.76, where S_VD_ and S_VG_ are the absolute noise intensities at V_D_ and V_G_, respectively. According to the empirical procedure of voltage-energy scale conversion used above, we have V_G_/V_D_ = ω_Γ_/ω_K_, therefore, the relation (1) becomes $({{\rm{S}}}_{{\rm{VD}}}/{{\rm{S}}}_{{\rm{VG}}}){(\langle {{\rm{g}}}_{{\rm{K}}}^{2}\rangle /2\langle {{\rm{g}}}_{{\rm{\Gamma }}}^{2}\rangle )}^{2}=1.76$. This relation allows the calculation of the matrix element ratio from the noise peak intensities. With the absolute values of S_VD_ = 9.4 × 10^−9^ V^2^/Hz and S_VG_ = 7.9 × 10^−9^ V^2^/Hz from Fig. 4 at the Kohn anomalies, the ratio of the matrix elements is: $\langle {{\rm{g}}}_{{\rm{K}}}^{2}\rangle /\langle {{\rm{g}}}_{{\rm{\Gamma }}}^{2}\rangle ={(7{{\rm{S}}}_{{\rm{VG}}}/{{\rm{S}}}_{{\rm{VD}}})}^{1/2}={(5.88)}^{1/2}=2.425$. Since the value of the matrix element ratio calculated from the noise data is strikingly close to the one obtained from the Raman spectrum, it results that electron-phonon coupling is the microscopic source responsible for the 1/f noise enhancement at Kohn anomalies. To our knowledge, this is the first experimental result which quantitatively supports electron-phonon coupling as microscopic source of 1/f noise in a solid-state system. The key factor in obtaining this result was the equation (1) deduced by Piscanec *et al*.^44^ for graphite only, hence the procedure is specific to carbon soot. Since the EPC is a fundamental physical mechanism in solid, the result we arrived at may raise the question whether it could act as microscopic source of 1/f noise in other solid-state systems. As shown above, phonon fine structure observed in different metals^37^ and metallic point contacts^35,36^ speaks in the favor of the phonon contribution to 1/f noise but the role of the matrix element remains to be elucidated. It is thus necessary to find other ways to go further. For instance, except for constant, a similar equation as (1) was deduced for two-dimensional silicene and germanene^53^, therefore it may be exploited to further investigate whether the procedure described above would apply to these materials, two. As for the semiconductors and, especially, semiconductor devices such an endeavor appears to be more complicated by the presence of other forms of noise spectra which coexist with the 1/f spectrum, such as, for instance, lorentzian 1/f^2^ or even 1/f^3^ spectra in very small devices^38^. A 1/f^2^ noise spectrum is generated by a mechanism of generation-recombination or the presence of random telegraph signal noise. Although such spectra usually manifest in the metal-oxide-semiconductor (MOS) transistor, as discussed in details in some work^54^, the presence of such spectra does not exclude the existence of a 1/f spectrum, their higher intensity merely obscures it. In search for the origin of 1/f noise in MOS transistor, such spectra should be eliminated, if possible, or devices with only 1/f noise should be selected. Image of the silicon phonon spectrum observed in the 1/f noise of MOS transistors^55^ is a hint that EPC could be involved in the 1/f noise of this device. In addition, if one considers^55^ that the tunneling into the interface/surface states is phonon-assisted (inelastic), then the correlation between the number of surface states and 1/f noise intensity can be explained in terms of electron-phonon interaction. That because more surface states means more tunneling events, hence more nonequilibrium phonons in the channel/interface generated by the inelastic processes. Consequently, “more surface states means more phonons in the channel and the interface, therefore, more noise”^55^. In principle, the omnipresence of electron-phonon interaction in solid and solid-state devices can be taken into account as argument in favor of EPC as source of 1/f noise, but it is only experiment which decides. Or, it was exactly this aspect which proved to be very difficult to solve for decades. In this work, we have reported the first quantitative example which supports the EPC as microscopic source of 1/f noise. Another qualitative one will be given later in this work for the case of graphene.

Standing for the local deviations from the linear dissipation law, the two noise peaks can now be ascribed to a nonlinear behavior of the matrix element at Kohn anomalies. Under the influence of the increasing voltage across the resistor terminals, the Fermi electrons transfer their energy to phonons which dissipate it with the group velocity dω/dk^56^. Since in the case of E_2g_-LO mode (G band) of graphite, Lazzeri *et al*.’s reported^52^ that the matrix element is proportional to the slope of the phonon dispersion, $\langle {{\rm{g}}}_{{\rm{\Gamma }}}^{2}\rangle$∝dω/dk, it results that a connection between the matrix element and the group velocity exists. Consequently, a nonlinear behavior of $\langle {{\rm{g}}}_{{\rm{\Gamma }}}^{2}\rangle$ requires nonlinear dispersion, such as the power dispersion law ω = βk^d^ (β−constant, d ≠ {0, 1}), which we assume to be valid at a Kohn anomaly. Due to the omnipresence of nonlinear terms in the current flow, the dc energy introduced by a battery in a resistor “is dissipated over a range of frequencies” or, alternatively, “over a range of wavenumbers”^45^ in the space domain. With this background idea, Teitler and Osborne^45^ calculated the fluctuation spectrum of the energy dissipated in a resistor biased at a dc voltage V. If the system is dispersive, k = k(ω), the spectral density in the space domain, S(k), can be translated into the frequency domain by the relation S(ω) = S(k)dk/dω. Using similarity and dimensional arguments from the theory of turbulent energy flow in fluids, they defined S(k) as S(k) ∝ η^6^(ηk)^ν^, where η is a characteristic length and (ηk)^ν^ is a dimensionless, “simple power low” similar to those encountered in the description of “turbulence, and ocean surface waves”^45^. With η∝(Δε)^1/5^, where Δε is the rate of energy dissipation per carrier mass density, they found S(k)∝(Δε)^(6+ν)/5^k^ν^. In the frequency domain, for the dispersion relation ω = βk^d^, S(k) becomes S(ω)∝(Δε)^(6+ν)/5^ω^−1+(ν+1)/d^. If one applies this formula to the case of a resistor biased at a dc voltage V, the condition of linear dissipation, S(ω)∼Δε∼V^2^, is obtained for ν = −1, while for ν ≠ −1, the general form of S(ω) is S(ω)∝(V)^2(6+ν)/5^/ω^1−(ν+1)/d^ = (V^2^/ω)[V^2/5^/ω^−1/d^]^ν+1^. Using the Hooge formula, one finally gets: ωS(ω)/V^2^ = α/N∝[V^2/5^/ω^−1/d^]^ν+1^. This relation shows that variations of ν around −1 attract deviations of the voltage exponent from 2 and, for a given d, of the frequency exponent from 1. Hence, the exponent ν + 1 = n may be considered as a nonlinearity parameter (n). This reappraisal of Teitler and Osborne nonequilibrium theory^45^ reveals that nonlinearity and dispersion affect both 1/f noise parameter and the frequency exponent. The relation α∝[V^2/5^ω^1/d^]^n^ suggests that the structure^33–41^ often observed in α is the signature of both nonlinear dissipation mechanisms and the interplay between nonlinearity and dispersion of the system. Moreover, the nonlinearity-dispersion interplay appears as the only factor which controls the behavior of the frequency exponent in the equation which results for it from the above analysis:

2 $${\rm{\gamma }}=1-n|d.$$

This new formula for the frequency exponent is very general in its simplicity. This simple equation predicts that, for a given *d*, any deviation of γ from 1 is due to nonlinearity. Resulting from a nonequilibrium theory^45^, the existence of this relation depends “on the presence of a nonlinear process”^44^. On the other hand, to explain Voss and Clarke^47^ fundamental experiment, namely why 1/f noise occurs in equilibrium, Tremblay and Nelkin^57^ introduced mode coupling terms (nonlinearity) in the transport equations and demonstrated that whatever small, nonlinearity is a *sine qua non* condition for 1/f noise to exist in thermal noise. This requires n≠0. If the nature of nonlinearity is the same in both equilibrium and nonequilibrium, the conclusion we are obliged to draw is highly disturbing: pure 1/f noise (γ = 1) might not exist in real systems! In these conditions, γ only tends to 1 for d»n. Naturally, Voss and Clarke stated that their “equilibrium measurements are […] obviously inconsistent with theories that rely on nonequilibrium processes”^47^. However, as shown above, from 1/f noise point of view, the two possible states of a physical system, equilibrium or nonequlibrium, must share a common property: nonlinearity. For instance, it is visible even in the work Voss and Clarke introduced the temperature fluctuation model of 1/f noise^58^. In this work, the authors reported heating-induced nonlinearities in the I(V) characteristic of a gold film. The noise spectrum of this film was measured at a voltage bias (0.81 V), which corresponds to the nonlinear regime, as results from the Fig. 3 of their work, and the film temperature was “as much as 40 C above room temperature”^58^. In their measurements, “the presence of heating nonlinearities indicated that the samples were much above the bath temperature”^58^, including those at liquid nitrogen and liquid-He. Therefore, nonlinearity in the film exists in this measurement but its possible role in the 1/f noise was not considered. Similar effects were observed by Eberhard and Horn^59,60^ in a silver film. They have found that the voltage exponent deviates from 2 when the sample starts heating at voltages higher than about 0.5 V. Nevertheless, the “values of [voltage exponent] greater than two seem to be present *even after the effects of sample heating are taken into account*.”^60^. The authors further concluded:”perhaps the most disturbing of our results is the deviation of the voltage exponent from the value 2.0 predicted by linear response theory.”^60^. Eberhard and Horn used equilibrium temperature fluctuation model to explain their result but found “sharp disagreement”^59^ with this theory. Therefore, although nonlinearity is a common factor in these works, its possible effect on noise mechanism was not investigated. Eberhart and Horn’s observations are of utmost importance for our findings because they suggest that besides heating-induced nonlinearity, another hidden, intrinsic source of nonlinearity could be involved in the generation of 1/f noise.

As shown in this work, electron-phonon coupling is the source of the nonlinearity responsible for the noise structure presented in Fig. 3. At the same time, one may suppose that the interplay between n and d in equation 1 could generate structure not only in α but also in the γ dependence on voltage. To verify this hypothesis, in Fig. 4 we show the dependence of the frequency exponent on voltage at an expanded scale. For comparison purposes, the absolute noise intensity is also presented. One notes that at this scale the faint wavy shape of the frequency exponent presented in Fig. 3 is, in fact, a structure which tracks the voltage dependence of the noise intensity not only at the Kohn anomaly but in the whole voltage range.

Such deviations from 1 in the spectral exponent are usually observed in its dependence on temperature, γ(T), in many solid-state systems and devices^10,12,61^ including carbon resistors^62^. Thermal activation model of Dutta, Dimon and Horn (DDH)^46^ quantitatively explains these dependences by the analytical relation γ = 1 − [∂lnS(ω,T)/∂lnT − 1]/ln(ω/ω_o_), where T is temperature, ω is the measurement frequency and ω_o_ is a frequency of the order of the phonon frequencies. For given ω and ω_o_, ln(ω/ω_o_) is a constant, therefore the deviations are dictated by [∂lnS(ω,T)/∂lnT − 1]. But, as was shown for semiconductors, metals^38^ and MOS transistors^55^ α is the image of the phonon density of states, α∼S(ω,T)∼F(ω), therefore (∂lnS(ω,T)/∂lnT∼[∂lnF(ω,T)/∂lnω][∂lnω/∂lnT], where ∂lnω/∂lnT stands for the lattice self-energy shift or nonlinearity (anharmonicity)^38^. It results that the nonlinearity is encoded in [∂lnS(ω,T)/∂lnT − 1], therefore, analogous to *n* in equation (2), this factor is a measure of nonlinearity and the exponent γ is “entirely determined by lattice specific parameters”^38^. In this form, DDH formula has been used to model existing temperature dependences γ(T) in MOS transistors^61^ and a silicon on sapphire film^63^ using the same, common structural factor: silicon phonon density of states^55^. Now, if one speculates that ω_o_ would not be a constant but, as previously, of the form ω_o_ = βk^d^, the factor ln(ω/ω_o_) becomes ln(k^−d^ω/β)∝-dlnk. Therefore, the ratio describing the deviations of γ(T) from 1 in the DDH equation has, apparently, the same physical significance as *n/d* in the equation (2). It results that at least qualitatively the two equations are closely related. Instead of S(ω,T) in DDH formula, some authors^64^ used α, once more emphasizing that, in fact, the DDH equation analytically describes an intricate connection between α and γ. In this respect, the results presented in Fig. 4 may create the impression that it is only the nonlinearity in α that reflects in the exponent. Existing experimental data show that the exponent itself induces structure in α. For instance, when the effect of the temperature dependence of γ on the 1/f noise parameter of silver and copper^59^ was took into consideration, thresholds in the noise magnitude were found at temperatures which correspond (k_B_T, k_B_−Boltzmann constant) to the fundamental phonon energies and their combinations^37^. It turns out that phonons are implied in the noise mechanism even when the sample is temperature scanned.

At microscopic level, the balance nonlinearity-dispersion in equation (2) can be understood by the same approach Akimenko, Verkin and Yanson^36^ used to explain the noise structure in sodium point contacts. Investigating the interaction between the Fermi surface and phonon dispersion curves in sodium, they associated the peaks and deeps in the 1/f noise at different voltages across the point contacts with the emission of Umklapp and normal phonons, respectively. This is because when gradually increasing the voltage, the electrons on Fermi surface probe the structure of the phonon branch they interact with at different wavenumbers and, consequently, “feel” any irregularity in their dispersion and/or the anisotropy in the electron-phonon coupling, as was shown to be the case at the Kohn anomalies. *Sensu stricto*, for a given voltage, the exact values of n and d in the equation (2) are dictated by the shape of both Fermi surface and the phonon branch at their intersection. If not impossible, an *in situ* measurement seems to be extremely difficult. A possible simpler way to find *n* would be to inspect I-V characteristics, but at very low or low voltages local deviations in it could be difficult to observe, even if the measurement is done at low temperature and the first or second derivative were used. For the determination of dispersion one has to resort to specific methods of lattice dynamics investigation. All these measurements-related aspects warrant further investigations. Due to the high uncertainties in the determination of both *n* and *d*, testing the validity of equation (2) is difficult. Nevertheless, if one considers the unique property of the dispersion exponent *d* to change its sign at a Kohn singularity, Eq. 2 can be used to predict the evolution of γ at such anomaly. Therefore, for a given value of the nonlinearity parameter *n*, due to the sign changes of *d* one might expect a transition around 1 in the frequency exponent at each anomaly. The necessary condition for noise intensity to increase with the voltage at Kohn anomalies (see Fig. 4) is *n* > 0. From Eq. (2), *n* = *d*(1 − γ) can be positive either for γ < 1 and *d* > 0 or γ > 1 and *d* < 0. On the other hand, we attributed the noise increase at a Kohn anomaly to a strengthening of the EPC. It can happen only if Fermi electrons interact with the anomalous, soft branch of the Kohn anomaly, which corresponds to *d* < 0. Consequently, if *n* > 0, a change from *d* > 0 to *d* < 0 should produce a transition from γ < 1 to γ > 1 at the Kohn anomaly. This prediction is confirmed by the data in Fig. 4, where sharp transitions (T) from γ < 1 to γ > 1 are clearly visible at both noise peaks. A transition from the anomalous (*d* < 0) to *d* > 0 phonon branch of the anomaly would correspond to a weakening of the electron-phonon coupling, therefore to a noise intensity decrease. For *d* > 0 and γ > 1, we get *n* < 0, which is the condition for noise intensity to decrease, as observed in Fig. 3 at the K (160 mV) point only. From this analysis, it may be inferred that, in general, the interplay between *n* and *d* at the intersection of the Fermi surface with a phonon branch can generate a structure in both α and γ. In the particular case of a Kohn anomaly, the strengthening-weakening of the EPC at it may result in the observation of the phonon spectrum in the electron conductivity fluctuations on Fermi surface. According to this interpretation, the noise curves reported in Figs 3 and 4 could be considered as the image of the phonon spectrum in the Fermi surface, a reciprocal effect to the one reported by Kohn^43^.

**Figure 5:**
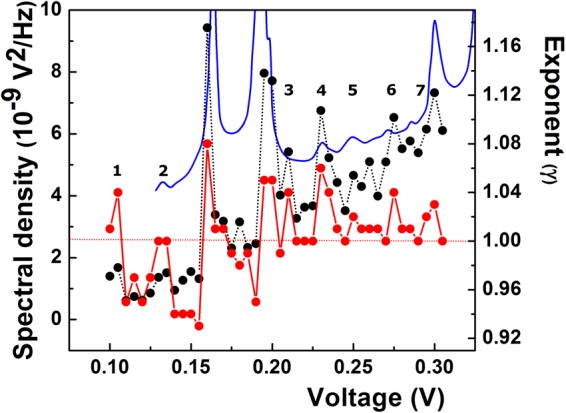
Comparison between the noise data (black dots - absolute spectral density; red dots - frequency exponent) and the Raman spectrum of graphene (blue curve - reproduced (adapted) with permission from ref.^75^, E. J. Heller et al. Theory of graphene Raman scattering. ACS Nano. 10, 2803-2818, 2016; Copyright 2016 American Chemical Society). Numbers denote the noise peaks assigned in Table 1. The energy scale of the Raman spectrum (cm^−1^) was transformed with the relation 8.06 cm^−1^ = 1 meV.

So far, our discussion was limited to the noise mechanism at Kohn anomalies. However, Fig. 4 shows that, besides anomaly-related structure, a weaker one develops in both α and γ, in the whole voltage range of our measurements. We should admit that it is very tempting to consider that it may be due to some spurious or random effect, such as temperature fluctuations, for instance. Although the correlation between α and γ in Fig. 4 points to the same common source, as shown previously, thermal effects in the resistor may contribute to this structure, especially in the case of γ. In search for the thermal heating as a possible source of nonlinearity in resistor, we calculated the resistance at each voltage point in the I-V characteristic (Fig. 2b) and found the same value (10.7 kΩ) in the whole voltage range. This indicates that heating-induced nonlinearity in our resistor is negligible. Next, the temperature of the resistor was calculated at different voltages. At the highest voltage across the resistor terminals (0.3 V), the current is 27.94 × 10^−6^ A. If the mass of carbon soot is m = 1 mg, with carbon specific heat c = 720 J/kgK and a measurement time for spectra acquisition of 15 seconds, the temperature increase in the resistor is ΔT = 0.17 K, while for 100 mV (the first point in our measurement) ΔT = 0.02 K. At the voltage of the first Kohn anomaly (0.160 V, 14.87 × 10^−6^ A), we get ΔT = 0.049 K, while at the second one ΔT = 0.07 K. Such insignificant increase in the temperature cannot explain almost an order of magnitude noise increase at the first Kohn anomaly, for instance. Therefore, Joule heating can be hardly considered as the source of the local nonlinear manifestation reported in Fig. 4 in both noise intensity and the spectral exponent at Kohn anomalies, at least. On the other hand, the unexpected correlation between α and the spectral exponent presented in Fig. 4 can be accounted for by dimensional considerations in the Hooge equation. As shown recently^65^, to keep this equation dimensionally correct, deviations of the voltage exponent from 2 must attract deviations from 1 in the frequency exponent. It turned out that, regardless of the nature of the excitation factor (voltage, temperature), any deviation of γ from 1 should be regarded as  signature of nonlinearity. This is exactly what Eq. (2) predicts. Consequently, if the source of nonlinearity is in the electron-phonon coupling, as shown in this work, then the structure in γ must be phonon-related. So far, we have shown that this is valid at Kohn anomalies only. However, besides the two dominant noise peaks at Kohn singularities,  two other significant noise peaks exist in Fig. 4 (see, also, Fig. 5) at 105 mV and 230 mV, respectively. Although not very well defined, the peak at 105 mV (1 in Table 1) could be the signature of another Kohn anomaly which manifests at Г, at about 102.3 meV^66^ or 107.6 meV^67^. By contrast, this is due to an out-of-plane optical phonon mode (ZO)^66^. To clarify the origin of the peaks, we further compared our noise data with those of Back *et al*.^41^ for metallic carbon nanotubes and found that besides the G-band-associated noise peak at about 200 mV, another clear, well-defined and dominant noise peak (resonance) is present in their noise data at 230 mV (ref.^41^, Fig. 2d). This is exactly the peak we found at 230 mV (4 in Table 1), which is assigned to a combination ZA + TO (230 meV) between an out-of-plane acoustic mode (ZA) and an in-plane transversal mode (TO), both at the M point of symmetry^68,69^. Other five noise peaks develop at (130–135)mV, 210 mV, 250 mV, 275 mV and 300 mV, denoted as 2, 3, 5, 6 and 7, respectively, in the Table 1. One mentions that only peaks having clear correspondent in the spectral exponent have been selected for comparison with the phonon energies.

**Table 1:** Comparison between noise peak voltages and phonon energies.

Peak no.	Peak voltage (mV)	Value from literature (meV or cm^−1^)	Assignment (Method of measurement)	Reference
1	105	102.3 107.6 104.95 meV (average)	ZO at Г (HREELS) ZO at Г (IXS) —	Politano^66^ Mohr^67^ —
2	130–135	134.4 (2 × 67.2)	2ZA at K (IXS)	Mohr^67^
3	210	210 (2 × 588 + 517 = 1693 cm^−1^)	2ZO + ZA at K (HREELS)	Yanagisawa^70^ (cited in Mounet^69^)
4	230	230 (465 + 1389 = 1854 cm^−1^)	ZA + TO at M (EELS)	Oshima^68^ (cited in Mounet^69^)
5	250	249.8 (2 × 124.9) 249.4 (3 × 670 = 2010 cm^−1^)	2TA at K (IXS) 3ZO at M (EELS)	Mohr^67^ Oshima^68^ (cited in Mounet^69^)
6	275	275 (3 × 530 + 627 = 2217 cm^−1^)	3ZA + ZO at K (HREELS)	Siebentritt^71^ (cited in Mounet^69^)
7	300	300(517 + 588 + 1313 = 2418 cm^−1^) 300 (57 + 3 × 81)	ZA + ZO + TO at K (HREELS) ZA + 3ZO at M (EELS)	Yanagisawa^70^ (cited in Mounet^69^) Oshima^68^ (cited inVitali^72^)

The values from literature in the column 3 are given either in meV or wavenumbers (cm^−1^). Wavenumbers are transformed in meV by the relation 1 meV = 8.06 cm^−1^. The significance of abbreviations in the column 4 is: Z- out-of-plane modes, otherwise in-plane modes; T-transversal, A-acoustic, O-optical; Г, K and M are points of symmetry in the graphite (graphene) Brillouin zone; HREELS-High Resolution Electron Energy Loss Spectroscopy; IXS-Inelastic X-ray Scattering; EELS-Electron Energy Loss Spectroscopy.

As can be seen in the Table 1, the noise peaks correspond to fundamental phonon modes in graphite or graphene (peak 1, ZO at Г^66,67^) or overtones, such as peaks 2 and 5 which are assigned to 2ZA at K^67^ and 2TA at K^67^ or 3ZO at M^68,69^, respectively. The combinations of overtones with another phonon are assigned to noise peaks 3 (2ZO + ZA at K^70^), 6 (3ZA + ZO at K^71^) and 7 (ZA + 3ZO at M^68,72^). Also, a three phonon combination (ZA + ZO + TO at K^69,70^) fits the noise peak 7. The fit between the noise peak voltages and the phonon energies in graphite/graphene is excellent. Very surprising in this correlation is the fact that, except maybe for the peak 5, all the weak noise peaks correlate with the out-of-plane phonons or combinations of these phonons with some in-plane modes. Spectroscopic observation of these phonons is notoriously difficult^73,74^ in graphene because, in sharp contrast with the in-plane phonons, the electrons interact very weakly with these phonons^74^. In graphene layers, the second-order overtones or combinations of these modes which develop “in the range of 1690–2150 cm^−1^”^73^ have been observed only recently. This wavenumber range is equivalent to (209–267)meV, which partially covers the voltage range where some less intense noise peaks are located. In fact, they are “forbidden in clean, perfect graphene crystals”^75^, so strong averages on samples with defects were necessary to unravel these “orphan phonons”^75^. Such a Raman spectrum, which is due to Bernard and coworkers^74^, is compared^40^ in Fig. 5 with our noise data. To this purpose, Fig. 4 was reproduced din Fig. 5, to avoid clutter.

Except for the peak 3 at 210 mV, the peaks in both noise intensity and spectral exponent track the phonon fine structure in the Raman spectrum. Also, the correlation extends to the noise peak 2. The noise peak 6 is slightly aside from its Raman counterpart, while a very weak, non-assigned noise feature at 285 mV is “visible” in the Raman spectrum. In passing, it corresponds to a combination 2ZA + LA (2 × 67.2 + 151 = 285.4 meV) at the K point^67^. As for the noise peaks 1and 2, a comparison (not shown in Fig. 5) with the phonon spectrum of graphite obtained by inelastic neutron scattering^76^ or the Eliashberg function of graphene obtained by inelastic tunneling spectroscopy^77^ gave an excellent correlation. These results indicate that the origin of weak noise structure is in the weak interaction between electrons and the out-of-plane phonon modes. The correlations presented above stand for a strong argument that, as in the case of Kohn anomalies, the origin of the peaks in both noise intensity and frequency exponent is in the electron-phonon coupling.

The forgoing discussion and the results presented in Figs 3–5 inherently point to a connection between α and the matrix element. In this respect, if one assumes the validity of the Hooge’s formula at both anomalies and using the Eq. (1), the ratio of the noise intensities becomes: α_D_/α_G_∝${(\langle {{\rm{g}}}_{{\rm{K}}}^{2}\rangle /\langle {{\rm{g}}}_{{\rm{\Gamma }}}^{2}\rangle )}^{2}$, where α_D_ and α_G_ are the mobility fluctuation parameters at voltages corresponding to D- and G-band, respectively. Hence, a generic connection ${\rm{\alpha }}\propto {(\langle {{g}}^{2}\rangle )}^{2}$ between α and the matrix element may hold. For instance, in graphene, theory of low field mobility^78^ due to optical phonons scattering (μ_L_) gives μ_L_∼1/〈g^2^〉, both at Г and K. According to the new relation for the 1/f noise parameter, we get $\alpha \propto {(\langle {{g}}^{2}\rangle )}^{2} \sim 1/{({{\rm{\mu }}}_{{\rm{L}}})}^{2}$, in agreement with the experiments which invoke phonon scattering as the microscopic source of 1/f noise^26^. On the experimental side, Zhang *et al*.^79^ observed that for single-layer graphene α∼1/(μ)^δ^, where δ = 1.5 and 3 for suspended and on-substrate structures, respectively. The authors normalized the original α values, so that all the data to fall on a single master curve. A closer examination of these data shows that for the suspended structures most of the α values on master curve follow a 1/(μ)^2.6^ dependence. Exactly the same dependence α∼1/(μ)^2.6^ has been reported for different InP-based two-dimensional electron gas structures with InGaAs channels^80^. We further examined whether the new correlation between α and the matrix element is able to explain the intriguing M-shape of the 1/f noise intensity vs. gate voltage observed both in single-^17,20^ and bilayer graphene^21^. To this purpose, we started from the Ando’s prediction that the G band frequency shift due to the electron-optical phonon interaction should feature “a logarithmic singularity when the Fermi energy is half of the energy of the optical phonon” for both mono-^81^ and bilayer graphene^82^. Such a G band phonon energy renormalization by the injected electrons has been observed experimentally both in mono-^83,84^ and bilayer graphene^85^. As predicted, they consist of two singularities (minima) located at Fermi energies equaling the half of the G band phonon energy, an approximate W shape with respect to the Dirac point. Transitions from phonon softening to stiffening which are expected at these anomalies (minima) should manifest as strengthening-weakening in the EPC, which is the strongest at the minima “because the energy of the electronic intraband excitations is exactly the phonon energy”^86^. According to the correlation ${\rm{\alpha }}\propto {(\langle {g}^{2}\rangle )}^{2}$, the strongest coupling at these phonon frequency minima would correspond to two maxima in α vs. gate voltage or Fermi energy. Consequently, the W-shaped dependence of the G band frequency on Fermi energy translates into an M-shaped dependence of (〈g^2^〉)^2^ and, accordingly, in α, on gate voltage, with respect to the Dirac point, as experimentally observed^17,20,21^.

The correlation between the noise intensity and the frequency exponent presented in Fig. 4 is highly intriguing. This is why we have investigated whether such correlations are visible in other physical systems. Unfortunately, detailed dependences of γ on voltage are not available, most of the existing investigations being focused on the temperature dependence of the two parameters. For instance, such detailed temperature dependences have been reported by Xiong *et al*.^61^ for metal-oxide-semiconductor (MOS) silicon transistors. We compared their data and found that γ and the noise intensity are strongly correlated in pre-irradiated, X-ray irradiated and post-irradiated annealed devices. It fact, the existence of such a correlation between γ and the noise intensity in a MOS transistor has been claimed for long^87^. In addition, same authors^87^ reported the time evolution of γ and the so-called noise energy (Fig. 5 from ref.^87^). Again, it is a matter of evidence that the evolutions of the two parameters are correlated in time. The presence of such correlated structures asks for a common microscopic source. In this respect, although the foregoing examples support our finding, they give no hint on the origin of this correlation. By contrast, as shown previously, the data presented in Figs 3–5 allowed the identification of EPC as the common underlying factor which controls both the noise intensity and the slope of the 1/f spectrum. However, at a first glance, how EPC would be able to modify the slope of the spectrum at low frequency does is not evident whatsoever. Nevertheless, in our view, such a manifestation of the EPC on the slope of the spectrum would be possible if the visible, low-frequency part of the spectrum extends into the thermal noise of the resistor till phonon frequencies. In other words, in order for EPC to affect the slope of the spectrum, 1/f noise should exist in thermal noise. So far, the existence of 1/f noise in thermal noise has been demonstrated by the fundamental experiment of Voss and Clarke^47^, later confirmed by Beck and Spruit^48^ by measurements in a carbonic material, only for systems in thermal equilibrium. The explanation we arrived at reveals the same facet of the 1/f noise for a system in nonequilibrium. Therefore, our finding seems to be a nonequilibrium analogue of the “1/f noise from systems in thermal equilibrium”^47^. It is very surprising that with this interpretation at hand, one can explain two fundamental hypotheses existing in the field of 1/f noise. For instance, Hooge procedure^25^ of thermal noise renormalization to deduce his formula, which asks for the presence of 1/f noise in thermal noise in nonequilibrium^65^, remained unexplained so far. Our finding offers the plausible physical justification for this empirical procedure. Moreover, our observation supports the hypothesis that the equipartition breakdown^65,88–90^ could be involved in the mechanism of 1/f noise. That because if the 1/f noise is hidden in thermal noise till phonon frequencies, it implies a classic-quantum transition at a given frequency, above which Planck’s blackbody radiation law is valid. Such a classic-quantum crossover in 1/f noise has been recently reported by Quintana *et al*.^91^.

## Conclusions

We reported 1/f noise peaks at the Kohn anomalies of carbon soot. A simple procedure was presented to calculate the ratio of the electron-phonon matrix element at the anomalies from the noise data. It compared very well with the one extracted from the Raman spectrum, which definitely identifies the electron-phonon coupling as the microscopic source of 1/f noise in carbon soot. Suggestions have been made on how to extend this result to other physical systems, such as silicene and germanene and even MOS transistor. We found that a fine interplay between nonlinearity and dispersion controls both the 1/f noise parameter and the frequency exponent. A new, general and simple formula was found for the frequency exponent, whose value is determined by the nonlinearity-dispersion balance. This formula revealed that, for constant dispersion, the deviations of the frequency exponent from 1 are the signatures of nonlinearity. The same conclusion we arrived at by dimensional considerations in the Hooge formula. Our analysis of the new formula revealed that nonlinearity is a *sine qua non* condition to have 1/f noise in both equilibrium and nonequilibrium. We have shown that nonlinearity and dispersion are hidden in the DDH formula which describes the temperature effect on the spectral exponent. It resulted that the two equations are related and both have in common phonon specific parameters. Exploiting the properties of the dispersion exponent at the phonon kink, this formula predicted transitions sublinear-supralinear in the frequency exponent at Kohn anomaly. This prediction was confirmed experimentally at both Kohn anomalies. It has been found that the dependence of the frequency exponent on voltage is featuring the same structure as the one observed in the noise intensity in the whole voltage range. The noise peaks, both in intensity and spectral exponent, have been correlated with the phonon energies in graphite/graphene in detail. Less intense noise peaks correlated very well with the out-of-plane phonon energies. It has been shown that the whole structure in noise intensity and spectral exponent is the image of the phonon spectrum. It turned out that the source of nonlinearity is in the electron-phonon coupling, which controls both the noise intensity and the slope of the spectrum. This effect was attributed to the extension of the 1/f noise spectrum in the thermal noise of the resistor till phonon frequencies. It stands for a nonequilibrium analog of the 1/f noise observed in systems in thermal equilibrium. This observation represents the long sought physical background for the Hooge empirical approach. Also, the violation of equipartition is another inherent consequence of our finding.
